# Association of ERCC family mutations with prognosis and immune checkpoint inhibitors response in multiple cancers

**DOI:** 10.1038/s41598-023-40185-7

**Published:** 2023-08-25

**Authors:** Chao Chen, Haozhen Liu, Yanlin Li, Jixian Liu

**Affiliations:** 1 Department of Thoracic Surgery, Peking University Shenzhen Hospital, Shenzhen Peking University-The Hong Kong University of Science and Technology Medical Center, Shenzhen, 518035 China; 2 Central Laboratory of Peking University Shenzhen Hospital, Shenzhen, 518035 China

**Keywords:** Cancer, Immunology, Biomarkers

## Abstract

The proteins encoded by the excision repair cross-complementing (ERCC) family are pivotal in DNA damage repair and maintaining genome stability. However, the precise role of the ERCC family in tumor prognosis and the effectiveness of immune checkpoint inhibitors (ICI) therapy remain uncertain. This study aimed to explore the connection between ERCC mutations and prognosis as well as the response to ICI. We observed that patients with ERCC mutations exhibited enhanced progression-free survival (PFS) and overall survival (OS) in two independent pan-cancer cohorts. Furthermore, this mutant subgroup showed higher tumor mutation burden (TMB) compared to the wild-type subgroup. Notably, ERCC mutations were associated with better OS (HR 0.54, 95% CI 0.42–0.70; P < 0.001) in pan-cancer patients who underwent ICI therapy (N = 1661). These findings were validated in a separate cohort, where patients in the ERCC mutant subgroup demonstrated improved clinical outcomes (HR 0.56, 95% CI 0.37–0.84; P = 0.03) and higher response rates (51.9% vs. 26.8%) than the wild-type subgroup. Further analysis revealed that patients with ERCC mutations displayed elevated tumor neoantigen burden (TNB) levels and increased infiltration of immune-response cells. Our study suggests that ERCC mutations are linked to enhanced immunogenicity and improved ICI efficacy, thus potentially serving as a biomarker for ICI therapy.

## Introduction

Immune checkpoint inhibitors (ICI) have shown significant improvements in clinical outcomes for patients with advanced or metastatic cancer^1^. ICI therapy is now a first-line treatment option for certain cancer types, such as metastatic melanoma, colorectal cancer, and non-small cell lung cancer (NSCLC)^2^. However, only a small proportion of patients respond positively to ICI therapy^3^. To identify patients who could potentially benefit from ICI treatment, the FDA has approved programmed death ligand-1 (PD-L1), high microsatellite instability (MSI-H), and tumor mutation burden (TMB) as biomarkers, but these are sometimes ineffective in predicting ICI responses. For instance, patients with negative PD-L1 or MSS status may still benefit from ICI treatment^4,5^. Additionally, researchers have identified tumor neoantigen load (TNB), intratumor heterogeneity, T cell-inflamed gene expression profile (GEP), and mutations in various cancer-related genes (such as *TP53*, *PTPRT*, KMT2 family, etc.) as supplementary predictors of ICI response^6,7^. Therefore, identifying biomarkers for ICI therapy is crucial for improving the efficiency of immunotherapy^8^.

Proteins encoded by the ERCC family (*ERCC1*, *ERCC2*, *ERCC3*, *ERCC4*, *ERCC5*, *ERCC6*, and *ERCC8*) are essential components of the complex nucleotide excision repair (NER) process^9^, playing a critical role in DNA damage repair and maintaining genome integrity^10,11^. For example, the proteins XPD and XPB, encoded by *ERCC2* and *ERCC3* respectively, act as helicase subunits of the transcription factor IIH (TFIIH) complex and are necessary for DNA damage verification^12,13^. After DNA damage verification, ERCC1 and XPF (encoded by *ERCC4*) form a heterodimer XPF-ERCC1, which, together with XPG (encoded by *ERCC5*), catalyzes the excision of DNA damage^14^. *ERCC6* (also known as CSB) and *ERCC8* (also known as CSA) are required for the assembly of the transcription-coupled NER machinery, which detects damage through its ability to sense transcription extension blockages^15^.

Numerous studies have suggested a close relationship between alterations in the ERCC family and the occurrence and development of cancer. For instance, several meta-analyses have demonstrated that single nucleotide polymorphisms of *ERCC2*, *ERCC3*, and *ERCC4* are associated with the risk of skin, breast, and lung cancers^16–18^. Li et al. found that *ERCC2* mutations increase cisplatin sensitivity in bladder cancer chemotherapy^19^, and studies by Stradella et al. showed that *ERCC3* mutations may elevate the risk of breast and ovarian cancer^20,21^. Additionally, the expression level of ERCC family genes has been linked to cancer prognosis. For example, Zhao et al. discovered that the expression level of *ERCC4* correlates with overall survival (OS) in ovarian and gastric cancers^22,23^. *ERCC5* downregulation has been identified as a biomarker for ovarian cancer prognosis and a potential therapeutic target^24^. However, the association between ERCC family mutations and ICI therapy response remains uncertain.

In this study, we analyzed available mutation and clinical data from public databases. Our findings revealed that patients with ERCC family mutations had a favorable prognosis in pan-cancer patients. Moreover, patients with ERCC mutations showed a higher TNB and increased infiltration of immune-response cells. Associations between ERCC mutations and improved ICI response were identified in the pan-cancer cohort, particularly in melanoma and NSCLC. Our study suggests that ERCC mutations may serve as biomarkers for tumor prognosis and ICI therapy.

## Results

### ERCC family genes were recurrently mutated in multiple cancer types

**Figure 1:**
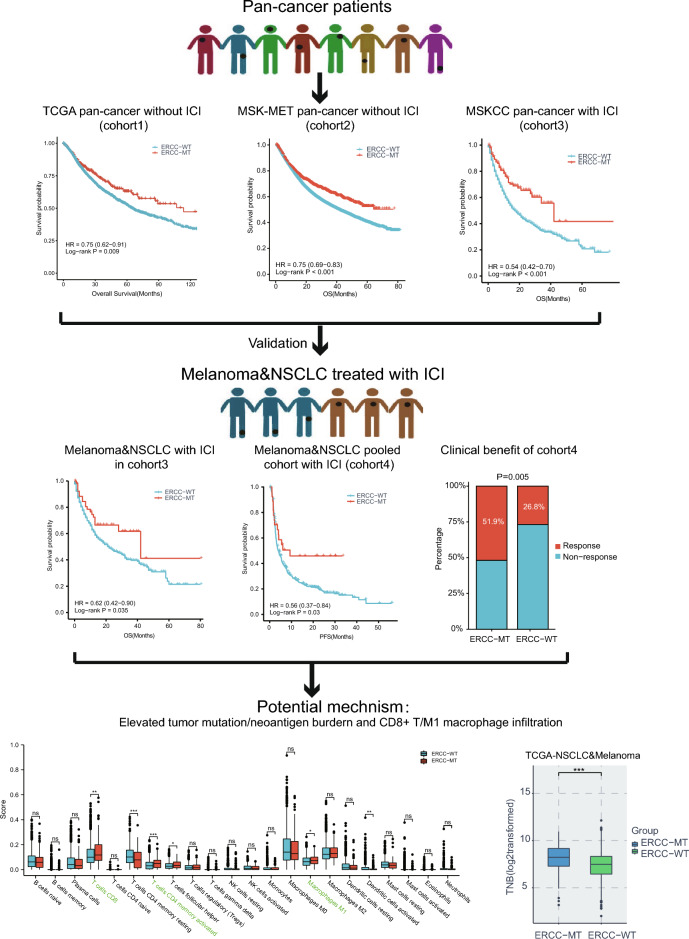
The workflow of this study.

**Figure 2:**
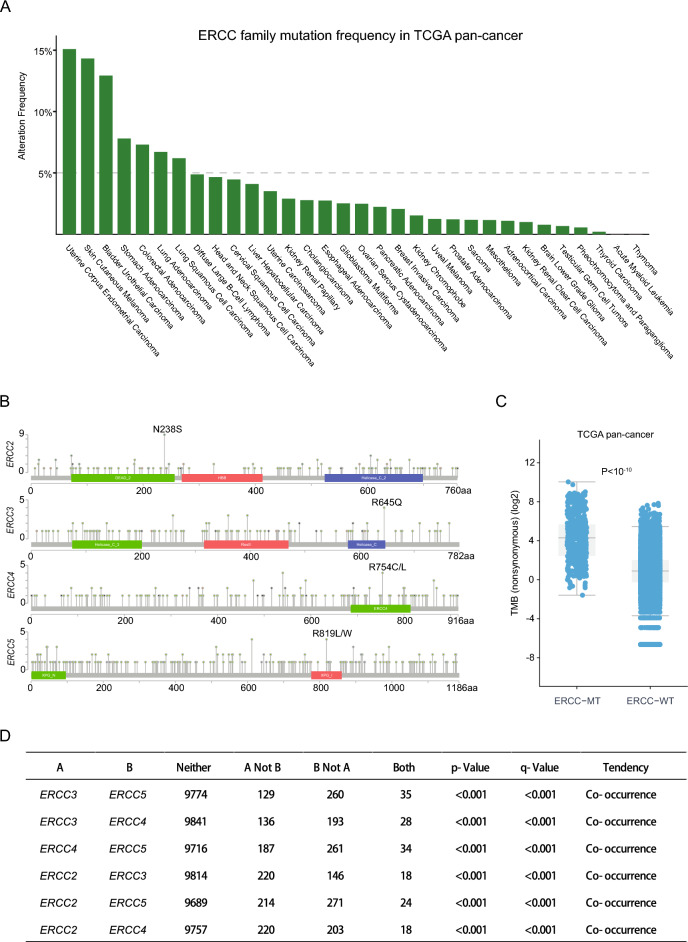
Frequency and mutation location of ERCC mutations among patients in the TCGA pan-cancer cohort are presented. (**A**) The frequency of ERCC mutations across 32 cancer types in patients who did not receive ICI therapy. (**B**) The information on protein domains and mutation locations for ERCC mutations. The color of the circle represents different non-silent mutation types. (**C**) The comparison of TMB between ERCC mutant and wild-type samples in pan-cancer. (**D**) The mutual exclusivity analysis reveals a tendency for co-occurrence of mutations among ERCC family genes.

Figure 1 shows the flow chart of this study. Initially, we investigated the association between ERCC mutations and cancer prognosis in a pan-cancer cohort, using data from the cBioPortal database. Our focus was on four core cancer-related genes of the ERCC family (*ERCC2*, *ERCC3*, *ERCC4*, *ERCC5*), as they are frequently altered in various cancer types, and are part of the MSK-IMPACT panel^25^. The accumulated mutation frequency of the ERCC family exceeded 5% in seven cancer types (Fig. 2A), including uterine corpus endometrial carcinoma, melanoma, bladder urothelial carcinoma, stomach adenocarcinoma, colorectal adenocarcinoma, lung adenocarcinoma, lung squamous cell carcinoma. Interestingly, no hotspot mutations were observed in the ERCC family genes (Fig. 2B), indicating that the mutations are primarily loss-of-function mutations.

Given that the ERCC family genes are primarily involved in DNA repair and play a crucial role in maintaining genome integrity, we sought to determine whether ERCC mutations are associated with TMB. As expected, the samples with ERCC mutations exhibited a higher TMB compared to the wild-type samples (Fig. 2C). Additionally, mutual exclusivity analysis on the cBioPortal database revealed a tendency for co-occurrence of mutations among ERCC family genes (Fig. 2D), emphasizing the relevance of mutations in these genes.

### The relationship of ERCC mutations with the prognosis of cancer

**Figure 3:**
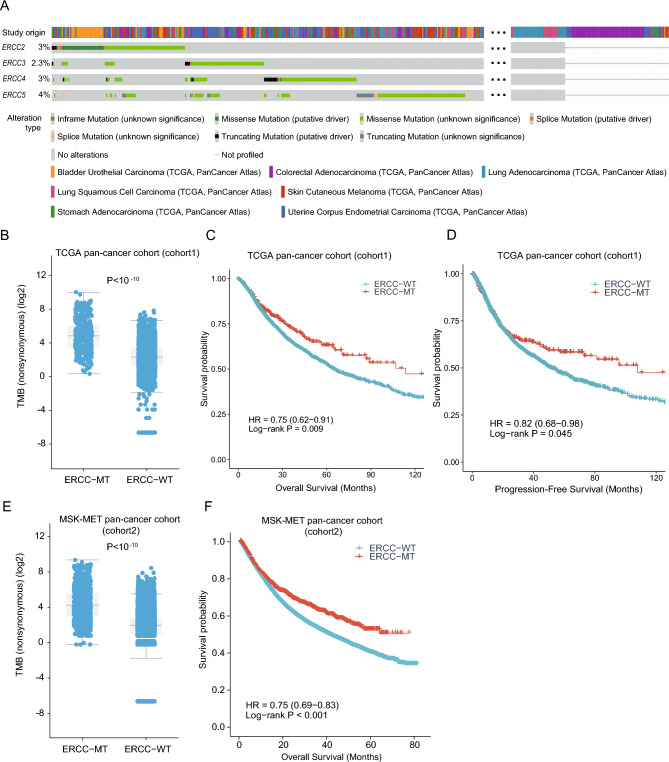
The impact of ERCC mutations on the survival outcomes of multiple cancer types was investigated. Firstly, we analysed the mutation patterns of ERCC family genes in seven cancer types, where the mutations exceeded 5% within the TCGA pan-cancer cohort (cohort1) (**A**). Next, we compared the TMB between the ERCC-mutant (ERCC-MT) subgroup and the ERCC-wildtype (ERCC-WT) subgroup in cohort1 (**B**). Subsequently, we assessed the overall survival (**C**) and progression-free survival (**D**) of patients in the ERCC-MT subgroup compared to the ERCC-WT subgroup in cohort1. Furthermore, we examined the TMB differences between the ERCC-MT subgroup and ERCC-WT subgroup in the MSK-MET pan-cancer cohort (cohort2) (**E**). Finally, we analysed the OS of patients in cohort2 based on ERCC mutation status (**F**).

The relationship between ERCC mutations and cancer prognosis was the next aspect of our investigation. For this analysis, we selected seven cancer types with a mutation frequency greater than 5% as cohort1 (Fig. 2A) to reduce background noise. The mutation frequencies were ranked as follows: *ERCC5*, *ERCC2*, *ERCC4*, and *ERCC3* (Fig. 3A). Consistent with previous findings, cohort1 samples with ERCC mutations exhibited higher TMB than the wild-type samples (P < 10E−10, Fig. 3B). Furthermore, we divided patients into two subgroups based on ERCC mutation status: the ERCC mutant subgroup (ERCC-MT) and the ERCC wild-type subgroup (ERCC-WT). The results indicated that mutations in the ERCC family were associated with improved OS and progression-free survival (PFS) in pan-cancer patients (HR 0.75, 95% CI 0.62–0.91; P = 0.009; Fig. 3C; HR 0.82, 95% CI 0.68–0.98; P = 0.045; Fig. 3D). To validate these findings, we further analyzed an independent pan-cancer cohort (cohort2). The results showed that samples with ERCC mutations still had higher TMB (P < 10E−10, Fig. 3E), and patients in the ERCC-MT subgroup exhibited better clinical outcomes compared to the wild-type subgroup (HR 0.75, 95% CI 0.69–0.83; P < 0.001; Fig. 3F).

### ERCC mutations linked with improved ICI efficacy in multiple cancer types

**Figure 4:**
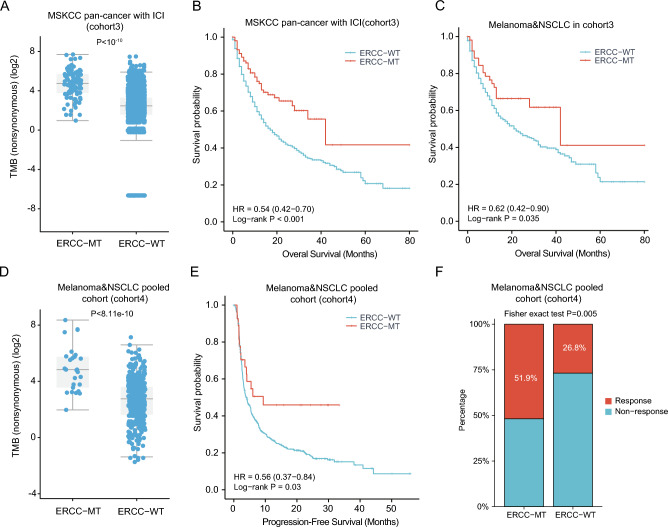
ERCC family mutations have been found to be associated with the clinical benefit of ICI therapy in various types of cancer. Here, we present the following analyses: (**A**) an evaluation of tumor mutational burden (TMB) between the ERCC-mutant (ERCC-MT) subgroup and ERCC-wild type (ERCC-WT) subgroup in cohort3. (**B**) Kaplan–Meier (KM) plot depicting the association of *ERCC* family mutations with the prognosis of patients in cohort3 who received ICI therapy. (**C**) KM plot focusing on melanoma and NSCLC patients in cohort3 who received ICI therapy. (**D**) Evaluation of TMB between the ERCC-MT subgroup and ERCC-WT subgroup in cohort4. (**E**) KM plot representing the survival outcomes of patients in cohort4 who received ICI therapy. (**F**) Clinical response analysis of patients in cohort4 who underwent ICI therapy.

Previous studies have demonstrated the association of certain cancer-related genes (e.g., *PTPRT*, *MUC16*, KMT2 family, FAT family genes etc.) with the response to ICI therapy^26–29^. Given the close involvement of ERCC family genes in DNA damage repair and the potential for ERCC mutations to result in higher TMB, we aimed to explore the relationship between ERCC mutations and ICI-treated response. To address this, we initially investigated the well-known pan-cancer cohort (cohort3, N = 1661) published by the MSKCC^30^. As anticipated, patients in the ERCC-MT subgroup exhibited a higher TMB than those in the wild-type subgroup (Fig. 4A). Moreover, the clinical outcome of patients in the ERCC-MT subgroup surpassed that of the ERCC-WT subgroup (HR 0.54, 95% CI 0.42–0.70; P < 0.001; Fig. 4B). Given that ERCC family genes are frequently mutated in melanoma and NSCLC, we further examined the impact of ERCC mutations on the prognosis of patients with melanoma and NSCLC within this cohort. The results revealed that patients in the ERCC-MT subgroup had more favorable survival outcomes compared to the ERCC-WT subgroup (HR 0.62, 95% CI 0.42–0.90; P = 0.035; Fig. 4C).

To ascertain the impact of ERCC mutations on the efficacy of ICI, we conducted an analysis in an independent cohort comprising 459 patients with melanoma and NSCLC who received ICI treatment (cohort4). Our investigation revealed that patients in the ERCC-MT subgroup exhibited a higher TMB compared to those in the ERCC-WT subgroup (Fig. 4D). Additionally, we observed similar results in terms of ERCC mutations correlating with improved survival outcomes (HR 0.56, 95% CI 0.37–0.84; P = 0.03; Fig. 4E). Moreover, a notable increase in the proportion of patients with clinical responses (complete response or partial response) was also evident in the ERCC mutated subgroup (51.9% vs. 26.8%, Fisher exact test P = 0.005; Fig. 4F).

### TNB and immune infiltration associated with ERCC mutations

**Figure 5:**
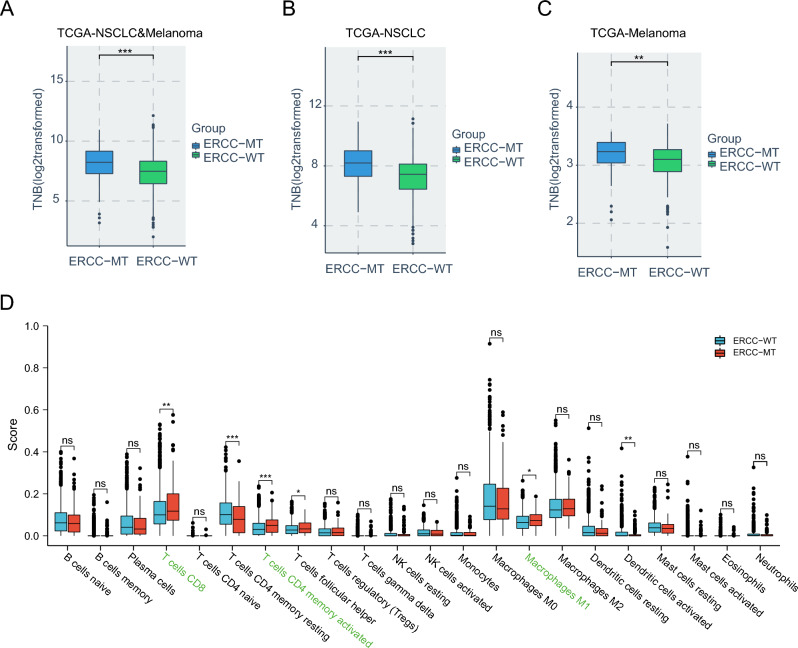
TNB and immune infiltration associated with ERCC mutations were evaluated as follows: (**A**) TNB comparison between the ERCC-MT subgroup and ERCC-WT subgroup in the TCGA NSCLC and Melanoma cohort; (**B**) TNB comparison between the ERCC-MT subgroup and ERCC-WT subgroup in the TCGA NSCLC cohort; (**C**) TNB comparison between the ERCC-MT subgroup and ERCC-WT subgroup in the TCGA Melanoma cohort; (**D**) Comparison of the infiltration scores of 22 immune cell types between the ERCC-MT and ERCC-WT subgroups (*p < 0.05; **p < 0.01; ***p < 0.001).

Tumor neoantigens play a critical role in the recognition of cancer cells by the immune system as they represent neoepitopes presented on the cell membrane by tumor cells^31–33^. To explore the potential mechanisms underlying ERCC mutations, we conducted analyses of TNB and immunological factors. Remarkably, our findings revealed a significant association between ERCC mutations and higher TNB in the TCGA melanoma and NSCLC cohort (Fig. 5A), indicating a correlation with increased tumor immunogenicity. Even when we separately assessed the TNB of the TCGA-NSCLC and TCGA-melanoma cohorts, the ERCC-MT subgroup still exhibited significantly elevated TNB compared to the ERCC-WT subgroup (Both P < 0.05; Fig. 5B,C). Moreover, the CIBERSORT algorithm analysis demonstrated an enhanced infiltration of CD8^+^ T cells and other pro-inflammatory immunocytes (e.g., activated memory CD4^+^ T cells, M1 macrophages) in the ERCC-MT subgroup (Fig. 5D). These cell types are known to be positive factors in the context of ICI therapy^34,35^.

## Discussion

ERCC family genes are known to play essential roles in the NER process and DNA damage repair^36^. Previous studies have suggested that ERCC genes may be involved in the tumorigenesis of various cancer types^22,23^. However, the relationship between ERCC genes and tumor prognosis has not been thoroughly investigated, and the association of ERCC mutations with ICI response remains uncertain. In this study, we observed recurrent mutations in ERCC genes in several cancer types, including melanoma, lung adenocarcinoma, lung squamous cell carcinoma, colorectal adenocarcinoma, and others. Interestingly, these mutations were found to be associated with a better prognosis in two independent pan-cancer cohorts. Additionally, ERCC mutations were linked to higher levels of TMB and TNB, indicating enhanced immunogenicity and potential favorable factors for ICI therapy. Notably, patients with ERCC mutations demonstrated improved clinical outcomes when treated with ICI, particularly those with melanoma and NSCLC.

We excluded *ERCC1* from our analysis for two main reasons. Firstly, the MSK-IMPACT panels used in clinical immunotherapy studies typically include several hundred oncogenes, tumor suppressor genes, and members of pathways actionable by targeted therapies. However, *ERCC1* was not part of all three different MSK-IMPACT panels (V1: 341 genes; V2: 410 genes; and V3: 468 genes), while *ERCC2*/3/4/5 were included in these panels^30,37^. Secondly, *ERCC1* has a lower mutation frequency compared to other ERCC family genes. Similarly, we did not include *ERCC6* and *ERCC8* in our study for the same reasons.

In recent years, TMB has gained recognition as a promising biomarker for immunotherapy^38,39^. However, several limitations, such as the absence of a certain threshold, the cost of whole exome sequencing, and variations in sequencing platforms and analysis pipelines, have significantly impacted the clinical application of TMB^40^. In contrast, evaluating the mutation status of one or a few genes is more straightforward and cost-effective. ERCC mutation status can be determined through targeted region sequencing, reducing sequencing expenses and simplifying the assessment of TMB and immunotherapy efficacy. Several research groups are already exploring the feasibility of using a small number of genes as markers for immunotherapy. For instance, Rui-Hua Xu et al. demonstrated that patients with *POLE* or *POLD1* mutations exhibited improved clinical outcomes with immunotherapy across various cancers^41^. Yue Yang et al. also revealed that the mutation status of *MUC4*, *MUC16*, and *TTN* was associated with the prognosis of gastric cancer and can be serve as a more economical and convenient marker for pan-cancer immunotherapy^27^.

The tumor microenvironment is another crucial factor associated with cancer prognosis and immunotherapy^42,43^. Previous studies have indicated that mutations in cancer-related genes may be correlated with the remodeling of the tumor microenvironment^29,44^. In this study, we observed that ERCC mutations were linked with increased infiltration of CD8^+^ T cells, along with other pro-inflammatory immunocytes (e.g., activated memory CD4^+^ T cells and M1 macrophages). These findings suggest that ERCC mutations are not only associated with heightened immunogenicity but may also contribute to a pro-inflammatory tumor microenvironment, which is more conducive to a positive response to ICI therapy.

This study has several limitations. Firstly, it is a retrospective study based on cohorts from previously published datasets, necessitating a prospective design for further validation. Secondly, the study includes several different cohorts comprising patients with different types and percentages of cancers. While these cohorts encompass several major cancer types (such as lung adenocarcinoma, lung squamous cell carcinoma, melanoma, colorectal adenocarcinoma, breast infiltrating ductal carcinoma, etc.), they may also introduce some bias in the data analysis. Additionally, the lack of experimental validation is another limitation.

In summary, through the integration of genomic alterations and clinical data, we have identified a potential association between ERCC mutations and the prognosis of multiple cancer types. Moreover, the mutation status of ERCC family genes is correlated with increased immunogenicity and improved clinical outcomes of ICI therapy, suggesting its potential as a biomarker for immunotherapy efficacy.

## Materials and methods

### Sample collection

#### Pan-cancer discovery cohort

The genomic alteration data (somatic mutations, including missense mutations, splice mutations, nonsense mutations, and indels) and clinical information on OS of TCGA samples across 32 cancer types were obtained by selecting “TCGA PanCancer Atlas Studies” from the cBioPortal database (https://www.cbioportal.org/, February 2022)^45,46^. Cohort1 (N = 3292) comprised seven cancer types with an ERCC family mutation frequency greater than 5%.

#### Pan-cancer validation cohort (cohort2)

A cohort consisting of 27 cancer types with over 25,000 samples from the Memorial Sloan Kettering—Metastatic Events and Tropisms (MSK-MET) dataset was used as a pan-cancer validation cohort (cohort2, N = 25,659)^47^.

#### Immunotherapy cohort

The somatic mutations and clinical annotation of ICI-treated pan-cancer data from 1661 patients (cohort3) at the Memorial Sloan-Kettering Cancer Center (MSKCC) were downloaded from the cBioPortal database (https://www.cbioportal.org/, February 2022)^30^. To further validate the predictive function of ERCC family mutations on ICI clinical response, we compiled a combined cohort of 459 ICI-treated patients (cohort4) from publicly available studies, comprising two cancer types: melanoma (N = 144)^48^, and NSCLC (N = 315)^49,50^. The clinical response information was obtained from the supplementary data of each study.

### Acquisition of mutation burden and neoantigen burden

Tumor mutation burden (TMB) was defined as the log2 transformation of the total non-synonymous mutations per megabase for samples conducted with whole exome sequencing (cohort1, cohort4). For the MSKCC cohort (cohort2, cohort3) with target region sequencing, TMB data were obtained from the cBioPortal database. The neoantigen data of melanoma and NSCLC samples in the TCGA were acquired from the Cancer Immunome Atlas (TCIA, https://www.tcia.at/home) database.

### Tumor-infiltrating immunocyte estimation

We utilized CIBERSORT to assess the abundance of 22 immunocyte subtypes, employing the LM22 signature^51^. The gene expression data of TCGA pan-cancer samples were obtained from the UCSC Xena database (https://toil-xena-hub.s3.us-east-1.amazonaws.com/download/tcga_RSEM_gene_tpm.gz). Subsequently, we used the gene expression data of NSCLC (containing lung adenocarcinoma and lung squamous cell carcinoma) and melanoma samples as inputs for the CIBERSORT algorithm.

### Statistical analysis

The Wilcoxon test was employed to determine the significance of the TMB and TNB comparisons among different subgroups. Kaplan–Meier survival curves were utilized to statistically analyze the OS and PFS data, and the log-rank test was employed to calculate the P value between the various subgroups. All statistical calculations were conducted using the cBioportal online database or R software (version 4.2.1). Visualization was carried out using the survminer package (version 0.4.9), and the statistical analysis of survival data was performed using the Survival package (version 3.2-10).

### Ethical approval

This study did not require ethical board approval because it did not contain human or animal trials.

## Data Availability

All data in this study were collected from the cBioPortal database (https://www.cbioportal.org), the UCSC Xena (https://pancanatlas.xenahubs.net), and TCIA database (https://www.tcia.at/home).

## Abbreviations

ICI: Immune checkpoint inhibitors
HR: Hazard ratio
TMB: Tumor mutation burden
TNB: Tumor neoantigen burden
ERCC: Excision repair cross-complementing
OS: Overall survival
PFS: Progression-free survival
